# Population waning trajectories of vaccine-induced tetanus immunity in Zhejiang, China

**DOI:** 10.3389/fimmu.2026.1824381

**Published:** 2026-05-25

**Authors:** Fuxing Chen, Yang Zhou, Yao Zhu, Xuejiao Pan, Linling Ding, Hanqing He, Xiaohua Qi

**Affiliations:** Institute of Immunization and Prevention, Zhejiang Center for Disease Control and Prevention, Hangzhou, China

**Keywords:** antibody, cross-sectional analysis, seroepidemiology, tetanus, tetanus vaccination

## Abstract

**Objective:**

Tetanus that occurs outside the neonatal period is not a statutory reporting disease in many regions, and few countries have comprehensive surveillance systems. To assess the level and durability of vaccine-induced tetanus immunity in a healthy population in Zhejiang, where long-term population-based data on non-neonatal tetanus immunity remain limited.

**Methods:**

A cross-sectional study was conducted in 2024 among 3,659 healthy individuals. Anti-tetanus toxoid IgG concentrations were measured by ELISA and expressed in IU/mL. Population immunity was evaluated by geometric mean concentration (GMC), seropositivity (≥0.01 IU/mL), and seroprotection (≥0.1 IU/mL). Antibody persistence was estimated according to age- and dose-specific antibody profiles. Generalized additive models (GAMs) with smoothing functions for the time since last doses were used to model nonlinear antibody trajectories overall, gender, and by dose group.

**Results:**

The overall Geometric Mean Concentration (GMC) of tetanus IgG antibodies was 0.064 IU/mL, with seropositivity (≥0.01 IU/mL) and seroprotection (≥0.1 IU/mL) rates of 85.05% and 52.94%, respectively. GMC peaked at 0.175 IU/mL in children aged 6–11 months, remained relatively high through 2 years of age, and then generally declined to 0.008 IU/mL in adults aged 40–59 years. By dose, GMC increased from 0.013 IU/mL in the 0-dose group to 0.156 IU/mL in the 3-dose group, then declined after 4 and 5 doses. Seroprotection exceeded 89% after 2–3 doses. GAM-fitted trajectories showed a rapid post-vaccination peak followed by gradual waning, with antibody levels remaining above 0.1 IU/mL for 8.81 years overall. Waning was slower in men than in women (7.87 vs. 6.66 years), and the estimated duration of seroprotection was 6.03, 12.86, and 3.07 years after three, four, and five doses, respectively.

**Conclusions:**

A high seropositivity rate does not necessarily indicate durable seroprotection against tetanus. Booster immunization strategies should incorporate both vaccination history and time since the last dose to sustain long-term protection.

## Introduction

Tetanus is a vaccine-preventable disease caused by *Clostridium tetani*. The amount of tetanus toxin that causes disease is insufficient to elicit natural immunity, recovery from the illness does not provide protection, and vaccination is the only way to gain long-term immunity (1, 2). The World Health Organization recommends 6 tetanus-toxoid-containing vaccine doses across the life course (3 primary doses plus 3 booster doses) at 12–23 months, 4–7 years, and 9–15 years, and routine adult booster vaccination is also recommended in the United States (2, 3). In China, the National Immunization Program provides 5 tetanus-containing doses including DTP at 3, 4, 5, and 18 months and a DT booster at 6 years during this study period, but routine booster vaccination beyond childhood is not included in the national schedule for the general adult population (4, 5).

Available evidence indicates that tetanus immunity declines with age and is influenced by vaccine formulation and booster dose history (6–8). In China, cross-sectional studies and a meta-analysis have described age-related declines in seroprotection, but the population-level life-course trajectory of tetanus antibody waning remains insufficiently characterized (6, 7, 9). This gap is important because prevention of non-neonatal tetanus in China remains challenging (10). In China, non-neonatal tetanus is not a notifiable disease and no dedicated surveillance system has been established, relevant data is still lacking to date, and the trajectory of population-level antibody dynamics remains poorly understood. Seroepidemiological surveillance provides a practical approach to evaluate community protection and identify immunity gaps (11). Therefore, we conducted a cross-sectional seroepidemiological study among healthy residents in Zhejiang Province to estimate the prevalence of protective tetanus antibody levels and to characterize the population waning trajectory of vaccine-induced tetanus immunity, with the aim of informing optimization of booster immunization strategies.

## Methods

### Study design and participants

In 2024, we conducted a community-based, cross-sectional serosurvey of an apparently healthy population in Zhejiang Province. All 11 cities in Zhejiang were included as surveillance sites. Eligible participants were apparently healthy residents aged 0–59 years, including both registered residents and non-registered residents who had lived locally for at least 6 months. Subjects were randomly selected from each city according to the following age groups: 1–5 months, 6–11 months, 12–17 months, 18–23 months, 2 years, 3 years, 4 years, 5 years, 6 years, 7–9 years, 10–14 years, 15–19 years, 20–39 years and 40–59 years, based on an age stratified sampling method. Each city was required to recruit at least 320 participants according to the provincial survey protocol, and approximately balanced sex composition within each age subgroup was sought whenever feasible.

### Sample size calculation

The sample size was calculated before the survey according to the provincial serological surveillance protocol. The required sample size for a cross-sectional survey was estimated using the formula: N=Z^2^[P(1-P)]/δ^2^*deff, where Z = 1.96 for a two-sided 95% confidence level; P = 0.30, representing a conservative anticipated seropositivity of 30% derived from the overall survey protocol; δ=0.022 was the allowable absolute error; and deff=2 was the design effect. Based on these assumptions, the minimum required sample size was 3334. After accounting for an anticipated non-response rate of approximately 5% and rounding to a multiple of the 11 cities, the final target sample size was set at 3520, corresponding to at least 320 participants per city.

### Questionnaire variables

Using a standardized interviewer-administered questionnaire, we collected demographic information, including date of birth, sex, household registration category, residence type (urban or rural), and occupation. The questionnaire also recorded the date of blood collection and immunization-related information, including the source of vaccination history (immunization information system, vaccination certificate, or participant/guardian recall), as well as the number and dates of vaccines containing tetanus components.

In the present tetanus-focused analysis, age was used to define the prespecified age groups and to estimate age-specific antibody levels and seropositivity. Sex and residence type were used to characterize the study population. Vaccination-related variables were used to reconstruct tetanus-containing vaccine dose history and the interval since the last documented dose for the waning analysis. Other questionnaire variables were collected for participant characterization and data-quality review and were not included in the primary generalized additive models.

### Reconstruction of vaccination history

Vaccination histories were reconstructed primarily from the Zhejiang Provincial Immunization Information System by linking questionnaire information and personal identifiers, including name, date of birth, and national identification number when available. The cumulative number of documented tetanus-containing doses before blood sampling was used to define vaccination dose categories, and the time since the last dose was calculated from the date of the most recent documented tetanus-containing dose to the date of blood collection.

The questionnaire also recorded the source of vaccination information (provincial immunization information system, vaccination certificate, or participant/guardian recall), which was used to assess the completeness of vaccination histories. To minimize misclassification, only verifiable documented doses with exact dates were used to determine dose category and time since the last dose; recall-only information was not used to reconstruct vaccination histories. For individuals vaccinated outside Zhejiang Province, records in the provincial registry may have been incomplete if earlier doses had not been transferred into the system. Therefore, participants with incomplete or unverifiable vaccination records were retained in the overall serological analyses but excluded from analyses requiring precise dose categorization or the interval since the last dose.

### Laboratory testing

Peripheral venous blood samples (3 mL per individual) were collected from participants and separated immediately on the collection day. Specimens were maintained under cold-chain transport and stored at −70 °C prior to laboratory testing. Laboratory tests were performed at Zhejiang Provincial Center for Disease Control and Prevention. Immunoglobulin G (IgG) antibodies were quantified by an enzyme-linked immunosorbent assay (Zhengzhou Yite Biotechnology Co., Ltd., China). The absorbance values were converted into antibody concentrations according to the standard curve. Results were reported in international units per milliliter (IU/mL). The cutoffs for protective antibody concentrations are 0.1 IU/mL for ELISA according to WHO recommendation (12).

### Handling of missing vaccination data

All participants with valid serological results were included in the overall analyses of tetanus IgG antibody concentrations, seropositivity, and seroprotection. Analyses stratified by vaccination dose category were limited to participants with clearly documented vaccination histories. Additionally, analyses of antibody waning since the last dose were further restricted to participants with system-documented dates of their most recent tetanus-containing vaccination. Missing vaccination records were not imputed.

### Statistical analysis

Antibody levels were presented as geometric mean concentrations (GMCs) with 95% confidence intervals (CIs). Seropositivity was defined by concentrations ≥0.01 IU/mL (basic protection), whereas seroprotection was defined by concentrations ≥0.1 IU/mL (adequate protection). To characterize the non-linear association between antibody levels and time since the last dose, generalized additive models (GAMs) were fitted to log-transformed IgG concentrations (13). Analyses of antibody waning in relation to the time since the last dose were restricted to participants with verifiable documented vaccination dates. The main model took the form: $\text{log}({\text{Y}}_{\text{i}})={\text{β}}_{0}+\text{s}(T_{\text{i}},\text{k})+{\text{β}}_{1}{\text{Sex}}_{\text{i}}+{\text{β}}_{2}{\text{Dose}}_{\text{i}}+{\text{ϵ}}_{\text{i}}$, where *T*_i_ denotes the time since the last dose (14). The GAMs were fitted using the mgcv package in R, with smoothing parameters estimated by restricted maximum likelihood. In the sex-specific analyses, models were stratified by sex category, whereas in the dose-specific analyses, models were stratified by dose category. Because current age and time since the last dose are structurally correlated, no concurrent adjustment was performed in any of the models. Model adequacy was assessed using residual plots, Q-Q plots, basis-dimension checks, and concurvity diagnostics. Antibody concentrations were log-transformed before group comparisons. Pairwise comparisons of GMCs among age groups and vaccine-dose groups were performed using the Games–Howell *post hoc* test, which is appropriate for unequal variances and unequal sample sizes and accounts for multiple pairwise comparisons. Statistical analysis was performed using R software, with a p-value threshold of <0.05 considered statistically significant.

### Estimation of threshold-crossing time

For each dose group, the fitted GAM was used to estimate the time at which the predicted GMC declined below the predefined seroprotection threshold. We report this as the model estimated threshold crossing time rather than a directly observed duration of protection. 95% CIs for these estimates were obtained using nonparametric bootstrap resampling with 1000 replicates.

### Ethical approval

Written informed consents were obtained from all participants or their legal guardians after the study’s purpose and methods were fully explained. The study protocol was reviewed and approved by the Ethics Review Committee of the Zhejiang Provincial Center for Disease Prevention and Control (CDC).

## Results

### Demographic characteristics of study population

A total of 3,665 individuals aged 0–59 years were enrolled in this survey. Of these, 6 were excluded due to poor blood sample quality, leaving 3,659 participants for subsequent analysis. As shown in **Table 1**, the study population included 1,885 males (51.52%) and 1,774 females (48.48%), with a male-to-female ratio of 1.06:1, and 1,973 urban residents (53.92%) and 1,686 rural residents (46.08%), with an urban-to-rural ratio of 1.17:1. Participants were classified into 14 age groups, with a mean age of 12.80 ± 14.91 years. Clearly documented tetanus-containing vaccine dose histories were available for 3,428 participants (93.69%), whereas 231 (6.31%) had unverifiable vaccination records. Verification rates were highest in younger age groups, reaching 100% among children younger than 2 years and remaining above 99% through most age groups up to 14 years. By contrast, unverifiable records were concentrated in older participants, particularly those aged 15–19 years (9.51%), 20–39 years (27.54%), and 40–59 years (27.59%).

**Table 1:** Baseline characteristics and data completeness.

Variable	Overall n = 3,659	Verified vaccination records n = 3,428	Unverified vaccination records n = 231
Age, mean ± SD	12.80±14.91	11.22±13.60	36.29±13.71
1~5 month	173	173 (100%)	0 (0.00%)
6~11 month	172	172 (100%)	0 (0.00%)
12~17 month	229	229 (100%)	0 (0.00%)
18~23 month	230	230 (100%)	0 (0.00%)
2 years	233	232 (99.57%)	1 (0.43%)
3 years	216	216 (100%)	0 (0.00%)
4 years	224	223 (99.55%)	1 (0.45%)
5 years	221	221 (100%)	0 (0.00%)
6 years	228	226 (99.12%)	2 (0.88%)
7~9 years	347	345 (99.42%)	2 (0.58%)
10~14 years	346	345 (99.71%)	1 (0.29%)
15~19 years	347	314 (90.49%)	33 (9.51%)
20~39 years	345	250 (72.46%)	95 (27.54%)
40~59 years	348	252 (72.41%)	96 (27.59%)
Sex			
Male	1885	1791 (95.01%)	94 (4.99%)
Female	1774	1637 (92.28%)	137 (7.72%)
Residence			
urban	1973	1879 (95.24%)	94 (4.76%)
rural	1686	1549 (91.87%)	137 (8.13%)
Vaccination dose group			
0	546	546	–
1	39	39	–
2	71	71	–
3	478	478	–
4	1173	1173	–
5	1121	1121	–

### Antibody level of tetanus IgG in healthy population

The overall geometric mean concentration (GMC) of tetanus IgG antibodies among the 3,659 healthy subjects was 0.064 IU/mL (95% CI: 0.062–0.067). As shown in **Table 2**, GMC was higher in males than in females [0.076 (95% CI: 0.072–0.080) vs. 0.054 (95% CI: 0.051–0.058) IU/mL] and higher in rural than in urban residents [0.071 (95% CI: 0.067–0.075) vs. 0.060 (95% CI: 0.056–0.063) IU/mL]. Age-specific analyses showed a schedule-related pattern in antibody levels. GMC was low in infants aged 1–5 months [0.031 (95% CI: 0.024–0.039) IU/mL], increased sharply to a peak of 0.175 (95% CI: 0.161–0.188) IU/mL at 6–11 months, remained relatively high at 12–17 months [0.151 (95% CI: 0.141–0.161) IU/mL], 18–23 months [0.162 (95% CI: 0.151–0.174) IU/mL], and 2 years [0.169 (95% CI: 0.160–0.177) IU/mL], and then generally declined with age to 0.008 (95% CI: 0.007–0.008) IU/mL in adults aged 40–59 years. A modest rebound was observed at 6 years [0.135 (95% CI: 0.121–0.150) IU/mL], consistent with the booster immunization schedule. Detailed pairwise comparisons of GMCs between age groups are provided in **Supplementary Table 1**.

**Table 2:** The tetanus toxoid IgG-specific antibody levels for healthy population in 2024.

Variables	Total (n)	GMC(IU/ml,95%CI)	Proportion		
			<0.01IU/ml,n (%)	0.01~0.1IU/ml,n (%)	≥0.1IU/ml,n (%)
Male	1885	0.076 (0.072, 0.080)	232 (12.30%)	553 (29.30%)	1100 (58.40%)
Female	1774	0.054 (0.051, 0.058)	315 (17.80%)	622 (35.10%)	837 (47.20%)
Urban	1973	0.060 (0.056, 0.063)	330 (16.70%)	680 (34.50%)	963 (48.80%)
Rural	1686	0.071 (0.067, 0.075)	217 (12.90%)	495 (29.40%)	974 (57.80%)
1~5 month	173	0.031 (0.024, 0.039)	69 (39.90%)	35 (20.20%)	69 (39.90%)
6~11 month	172	0.175 (0.161, 0.188)	3 (1.70%)	3 (1.70%)	166 (96.50%)
12~17 month	229	0.151 (0.141, 0.161)	1 (0.40%)	25 (10.90%)	203 (88.60%)
18~23 month	230	0.162 (0.151, 0.174)	2 (0.90%)	21 (9.10%)	207 (90.00%)
2 years	233	0.169 (0.160, 0.177)	1 (0.40%)	14 (6.00%)	218 (93.60%)
3 years	216	0.140 (0.126, 0.156)	6 (2.80%)	32 (14.80%)	178 (82.40%)
4 years	224	0.108 (0.095, 0.121)	7 (3.10%)	58 (25.90%)	159 (71.00%)
5 years	221	0.106 (0.096, 0.117)	2 (0.90%)	79 (35.70%)	140 (63.30%)
6 years	228	0.135 (0.121, 0.150)	3 (1.30%)	55 (24.10%)	170 (74.60%)
7~9 years	347	0.094 (0.087, 0.102)	2 (0.60%)	143 (41.20%)	202 (58.20%)
10~14 years	346	0.056 (0.051, 0.062)	16 (4.60%)	231 (66.80%)	99 (28.60%)
15~19 years	347	0.042 (0.038, 0.047)	43 (12.40%)	214 (61.70%)	90 (25.90%)
20~39 years	345	0.019 (0.017, 0.021)	116 (33.60%)	203 (58.80%)	26 (7.50%)
40~59 years	348	0.008 (0.007, 0.008)	276 (79.30%)	62 (17.80%)	10 (2.90%)

When stratified by dose group (**Table 3**), GMC increased markedly with the number of vaccine doses from 0.013 IU/mL (95% CI: 0.012–0.014) in the 0-dose group to 0.056 (95% CI: 0.036–0.085), 0.153 (95% CI: 0.128–0.177), and 0.156 (95% CI: 0.149–0.162) IU/mL in the 1-, 2-, and 3-dose groups, respectively, and then decreased to 0.123 (95% CI: 0.117–0.129) and 0.061 (95% CI: 0.058–0.065) IU/mL in the 4- and 5-dose groups, respectively. Detailed pairwise comparisons of GMCs between dose groups are shown in **Supplementary Table 2**.

**Table 3:** Distribution of tetanus toxoid IgG-specific antibody levels by doses.

Doses	Total (n)	GMC(IU/ml,95%CI)	Proportion[n(%)]		
			<0.01IU/ml,n (%)	0.01~0.1IU/ml,n (%)	≥0.1IU/ml,n (%)
0	546	0.013 (0.012, 0.014)	294 (53.80%)	215 (39.40%)	37 (6.80%)
1	39	0.056 (0.036, 0.085)	8 (20.50%)	10 (25.60%)	21 (53.80%)
2	71	0.153 (0.128, 0.177)	3 (4.20%)	3 (4.20%)	65 (91.50%)
3	478	0.156 (0.149, 0.162)	3 (0.60%)	47 (9.80%)	428 (89.50%)
4	1173	0.123 (0.117, 0.129)	31 (2.60%)	255 (21.70%)	887 (75.60%)
5	1121	0.061 (0.058, 0.065)	94 (8.40%)	563 (50.20%)	464 (41.40%)

### Seropositivity and seroprotection rates of tetanus IgG antibodies

**Figure 1:**
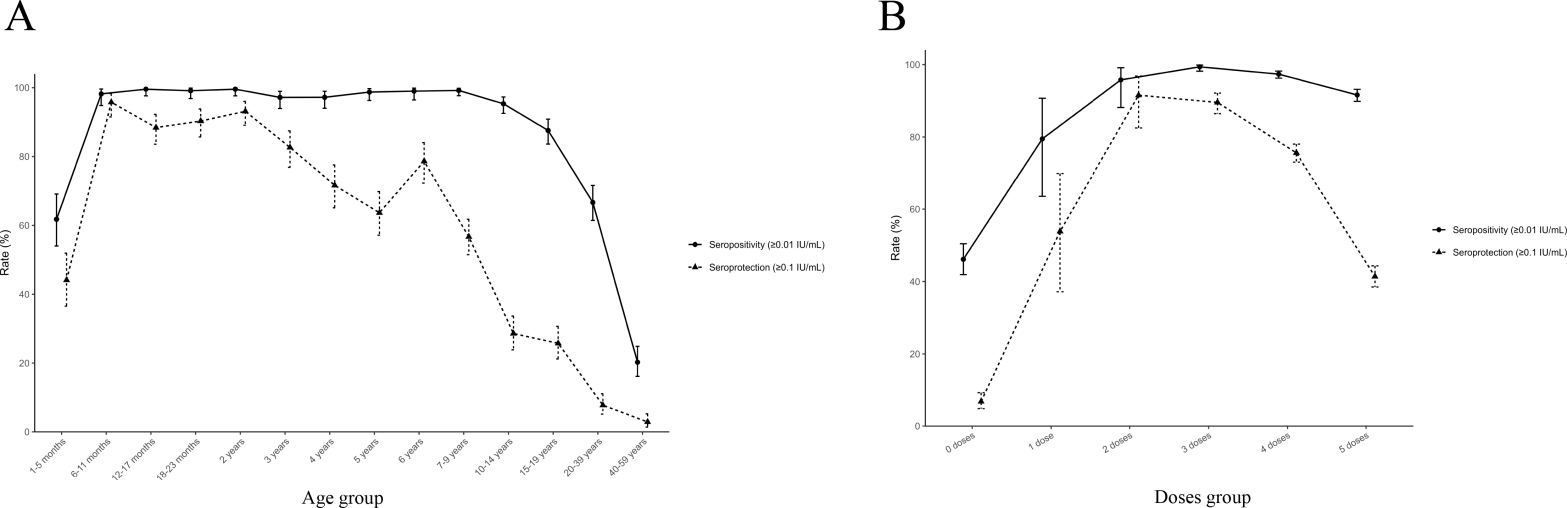
Seropositivity and seroprotection rates of tetanus IgG antibodies by age group and documented vaccine-dose group. Panel **(A)** shows the seropositivity and seroprotection rates across age groups in the study population. Panel **(B)** shows the corresponding rates according to the number of documented tetanus-containing vaccine doses among participants with verifiable vaccination records. The solid line with circles indicates seropositivity, defined as tetanus IgG concentration ≥0.01 IU/mL, and the dashed line with triangles indicates seroprotection, defined as tetanus IgG concentration ≥0.1 IU/mL. Error bars indicate 95% confidence intervals.

Of the 3,659 participants, tetanus vaccination dose history was clearly documented for 3,428. The overall seropositivity (≥0.01 IU/mL) and seroprotection (≥0.1 IU/mL) rates for tetanus IgG antibodies were 85.05% and 52.94%, respectively. As shown in **Figure 1**, seropositivity remained close to 100% throughout most of childhood, whereas seroprotection declined progressively with age and dropped sharply after 10 years (**Figure 1A**). Seropositivity was markedly higher in vaccinated participants than in those with 0 doses, rising from 46.20% in unvaccinated individuals to approximately 79.5% after 1 dose and 95.8% after 2 doses, and reaching 99.40% after 3 doses. Seropositivity remained high after 4 doses (97.4%) and was still 91.6% after 5 doses. Seroprotection showed a similar but not identical pattern, increasing from 6.80% in the 0-dose group to 53.8% after 1 dose and peaking at 91.50% after 2 doses; it remained high after 3 doses (89.5%) but declined to about 75.6% and 41.4% in the 4-dose and 5-dose groups, respectively (**Figure 1B**).

### The nonlinear relationship between the time since the last documented vaccine dose and tetanus antibody levels

**Figure 2:**
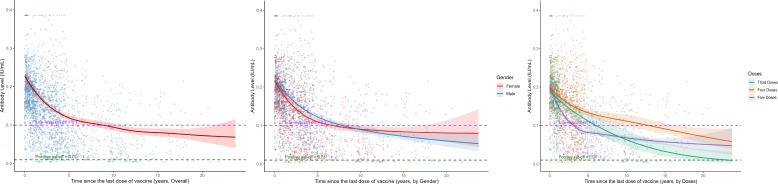
Generalized additive model estimates of tetanus IgG concentrations according to time since the last dose. Solid lines indicate model-predicted antibody concentrations, and shaded bands indicate pointwise 95% confidence intervals. The purple horizontal dashed line indicates the seroprotective threshold of 0.1 IU/mL. The green horizontal dashed line indicates the seropositive threshold of 0.01 IU/mL.

To evaluate tetanus antibody trajectories, we fitted generalized additive models (GAMs) to characterize the nonlinear association between time since the last documented dose and tetanus IgG concentrations (**Figure 2**). Antibody levels peaked shortly after vaccination and then declined gradually, remaining above the 0.1 IU/mL threshold for approximately 8.81 (95% CI:5.76-10.98) years overall. Sex-stratified analyses showed a slower decline in men than in women, with the fitted curves crossing the protective threshold at approximately 7.87 (95% CI:6.54-10.61) and 6.66 (95% CI:5.06-11.21) years, respectively. Dose-stratified analyses estimated times to decline to 0.1 IU/mL of approximately 6.03 (95% CI:4.20-13.13), 12.86 (95% CI:9.33-17.24), and 3.07 (95% CI:2.61-3.57) years after three, four, and five doses, respectively. Consistent with these patterns, **Supplementary Table 3** showed progressively longer intervals since the last dose and lower GMCs across the 3-, 4-, and 5-dose groups, together with declining seroprotection (90.04%, 76.70%, and 46.46%, respectively), despite persistently high seropositivity. The lower GMC in the 5-dose group compared with the 4-dose group was statistically significant (P<0.001), suggesting that the shorter estimated protection duration after five doses likely reflects group composition and time since vaccination rather than reduced vaccine effect. Concurvity diagnostics for the overall adjusted GAM are shown in **Supplementary Table 4**. The smooth term for time since vaccination showed low to moderate concurvity, with worst, observed, and estimate values of 0.266, 0.224, and 0.062, respectively. Model diagnostics supported an overall acceptable fit, with no major evidence of misspecification, only mild tail deviation in QQ plots and mild heteroscedasticity, and adequate basis dimension according to k-index and k-check results (**Supplementary Figure 1**-**S3**).

## Discussion

In this cross-sectional study of 3,659 individuals, we evaluated the real-world magnitude and persistence of tetanus IgG antibodies after vaccination in Zhejiang Province, China. The overall GMC of tetanus IgG antibodies was 0.064 IU/mL. Although seropositivity (≥0.01 IU/mL) remained high, only about half of the participants achieved the conventional seroprotective threshold (≥0.1 IU/mL). Given the cross-sectional design of this study, the inferred waning trajectories should be interpreted as population-level estimates rather than direct observations of intra-individual antibody decay. Despite this limitation, the marked decline in seroprotection following childhood indicates that booster immunizations may be needed to maintain population immunity.

The overall seropositivity rate of tetanus antibody in this study was 85.05%, which was lower than the 99.97% reported in the Xinjiang Uygur Autonomous Region in 2023 (15), and slightly higher than the 84.39% in Shaanxi Province and the 76.48% in Henan Province from 2022–2023 (16, 17). This discrepancy may reflect differences in detection reagents and laboratory testing methods, and may be likely due to regional differences in tetanus toxoid–containing vaccine (TTCV) coverage. The positivity rate in this study was lower than that reported in the United States (93.8%) (18) and Greece (92.34%) (19), which may be attributable to differences in national immunization programs and public health initiatives. In contrast to the relatively high seropositivity rate, the seroprotection rate in our study was only 52.94%, which was higher than that reported in Henan (41.72%) (17) and Jiangsu (29.00%) (20), but lower than that reported in Greece (59.5%) (19). Men had higher seropositivity and seroprotection rates than women, consistent with previous studies (20, 21). One possible explanation is that men are more frequently engaged in physically demanding or occupational exposure, increasing the likelihood of tetanus vaccination after trauma.

The overall GMC of 0.064 IU/mL was similar to that reported in the healthy population of Henan Province (0.067 IU/mL) but notably lower than that observed in the Italian general population (0.63 IU/mL) (22). Such differences may partly arise from national immunization strategies. Italy implements a life-cycle vaccination program that includes an adolescent dTap booster at 12–18 years of age (23) and decennial boosters for adults (24), thereby helping to maintain higher population immunity. In contrast, tetanus booster vaccinations are generally not provided after 6 years of age in China (25), resulting in waning immunity among adolescents and adults. Post-exposure prophylaxis practices are a plausible source of differences in serum antibody levels. In China, wound management often prioritizes passive immunization with tetanus antitoxin (TAT) or tetanus immunoglobulin (TIG) (26), whereas active boosting with tetanus toxoid–containing vaccines is used more systematically in many European settings (27). Passive immunization provides only temporary protection and does not induce immunological memory or durable antibody production (28), these differences may contribute to lower long-term antibody levels.

Age was strongly associated with antibody level in our study. Higher antibody concentrations were observed in younger children, whereas lower levels are found in older individuals. Among population younger than seven years, the proportion of protective antibodies (≥0.1 IU/mL) remained high, with a slow rate of decline. After an initial peak of 0.173 IU/mL at 6 months, the GMC declined to 0.147 IU/mL at 12–17 months and then increased to 0.168 IU/mL by 2 years old after booster vaccination. This pattern likely reflects the durable protection induced by the primary series and early booster doses, potentially reinforced by exposure to conjugate vaccines that use tetanus toxoid as a carrier protein (12). A relative plateau was observed at 7–9 years of age, in which the proportions of seropositivity and seroprotection were similar. Thereafter, seroprotection declined sharply after 9 years of age and reached its lowest level in the 40–59 age group. This finding is consistent with previous reports showing age-related waning of tetanus immunity (29, 30).

Our findings are also noteworthy in light of current WHO recommendations. WHO recommends that lifelong protection requires 6 doses of tetanus-toxoid-containing vaccine (3 primary doses and 3 booster doses), with booster doses preferably administered during the second year of life, at 4–7 years, and at 9–15 years old (2). By contrast, the routine schedule in China includes 5 childhood doses, with the last routine booster administered at 6 years of age (25). Therefore, the decline in seroprotection observed in our study after age nine reflects the immunity gap targeted by the WHO-recommended adolescent booster. These findings support the need to further evaluate booster strategies for older children, adolescents, and adults in China.

The antibody waning trajectories following vaccination is a critical factor in understanding the dynamics of population-level immunity. Antibody waning trajectories may also differ by sex. In our fitted population-level curves, the estimated time to decline to the seroprotective threshold (0.1 IU/mL) was shorter in females than in males (approximately 6.66 vs. 7.87 years). The corresponding 95% confidence intervals based on bootstrap overlapped (females: 5.06–11.21 years; males: 6.54–10.61 years), indicating considerable uncertainty around this difference. This pattern is broadly consistent with recent seroepidemiological studies reporting lower tetanus seroprotection or antibody levels among females, particularly in older age groups (18, 22, 31). Sex and gender differences in vaccine responses had been described in the literature, potentially involving hormonal, genetic, and immunoregulatory influences (32, 33). However, these mechanisms were not measured in the present study and should therefore be regarded as hypotheses rather than conclusions.

In this study, we also observed a dose-persistence mismatch phenomenon. The estimated duration of seroprotection varied according to the number of doses administered, with protection lasting approximately 6.02 (95% CI: 4.20-13.13) years following three doses, 12.86 (95% CI: 9.33-17.24) years after four doses, and 3.07 (95% CI: 2.61-3.57) years after five doses. As shown in **Supplementary Table 3**, dose group was strongly associated with age and time since the last vaccination. Participants in the 3-, 4-, and 5-dose groups had mean ages of 1.42, 4.56, and 10.73 years, respectively, and mean intervals since the last dose of 0.89, 2.89, and 4.56 years, respectively. Thus, the 5-dose group largely represents children sampled several years after the DT booster administered at 6 years of age, whereas the 4-dose group includes younger children who were sampled closer to recent vaccinations. In addition, uptake of non-NIP vaccines such as DTaP-IPV and DTaP-IPV-Hib has increased in Zhejiang (34, 35), and some children may also have received conjugate vaccines containing tetanus toxoid as a carrier protein, such as Hib, PCV13, and group A/C meningococcal conjugate vaccines (12, 35). These additional exposures may have enhanced antibody responses in some children, particularly in the younger dose groups.

Although the GMC was lower in the 5-dose group than in the 4-dose group, seropositivity remained high in both groups, whereas seroprotection declined substantially. This suggests that many children in the 5-dose group still had detectable antibodies but had fallen below the 0.1 IU/mL threshold. Therefore, the apparently shorter persistence after five doses should not be interpreted as evidence that the fifth dose biologically impairs long-term immunity. Rather, it is more likely to reflect threshold crossing, longer time since the last dose, and heterogeneity in additional vaccine exposures. Previous studies evaluating immune persistence after booster vaccination in children have reported that most participants remained seroprotected for about 3.5 years after the booster dose (36, 37), which is broadly consistent with our estimate for the 5-dose group.

Several limitations should be considered in this study. Our study was based on a cross-sectional study, causal inference regarding antibody persistence is limited, and the estimated waning trajectories represent population-level patterns rather than individual longitudinal changes. Comparisons across dose groups are susceptible to residual confounding by age, time since the last vaccination, receipt of post-exposure boosters, and use of self-paid non-NIP vaccines. Furthermore, incomplete vaccination records may have introduced information bias. Because China does not yet have a fully unified national immunization information system, vaccinations administered outside Zhejiang Province or through self-paid channels may not have been captured, potentially leading to misclassification of vaccination status. Future longitudinal studies with complete vaccination histories, including non-NIP vaccines and post-exposure boosters, are needed to more precisely define the durability of tetanus immunity.

In conclusion, this study delineates the population-level trajectory of tetanus antibody levels from birth to adulthood in a healthy community-based population. Seroprotection was more sustained in children than in adults, while antibody levels in adulthood declined progressively over time. Higher numbers of vaccine doses were associated with longer antibody persistence, underscoring the importance of complete and sustained immunization across the life course. Although sex-stratified analyses suggested possible differences in antibody waning, these findings should be interpreted cautiously. Overall, our results enhance the understanding of long-term tetanus antibody dynamics and may help inform age-appropriate and targeted booster strategies to maintain durable population immunity.

## Data Availability

The original contributions presented in the study are included in the article/**Supplementary Material**. Further inquiries can be directed to the corresponding author.

## Supplementary material

Supplementary Table 1 Pairwise *post hoc* comparisons of tetanus IgG geometric mean concentrations across age groups.

Supplementary Table 2 Pairwise *post hoc* comparisons of tetanus IgG geometric mean concentrations across documented vaccine dose groups.

Supplementary Table 3 Age distribution, GMC, sex, residence, time since the last dose, seropositivity, and seroprotection by dose group based on system verification vaccination.

Supplementary Table 4 Concurvity diagnostics for the overall adjusted GAM.

Supplementary Figure 1 Diagnostic plots for the overall adjusted generalized additive model (GAM). The plots show the quantile-quantile plot of deviance residuals, residuals versus the linear predictor, the histogram of residuals, and observed versus fitted values. Red reference lines indicate the expected pattern under adequate model fit.

Supplementary Figure 2 Basis-dimension diagnostics for the overall adjusted generalized additive model (GAM). The plots show the smoothing criterion, EDF saturation, minimum k-index, and minimum k-check P value across candidate basis dimensions (k). Red dashed lines indicate reference thresholds.

Supplementary Figure 3 Residual diagnostics for the overall adjusted generalized additive model (GAM). The plots show residuals versus fitted values, the QQ plot of deviance residuals, the scale–location plot, and observed versus fitted values. Blue smooth curves indicate local trends, and red dashed or solid lines indicate reference lines.
