# Supplementary material for: Population waning trajectories of vaccine-induced tetanus immunity in Zhejiang, China
Source: Front Immunol. 2026 May 25;17:1824381. doi: 10.3389/fimmu.2026.1824381 (PMC13243430; doi:10.3389/fimmu.2026.1824381)
Supplement: Supplementary Table 1 — Pairwise post hoc comparisons of tetanus IgG geometric mean concentrations across age groups. [file SupplementaryFile1.docx]

Supplementary Table S1. Pairwise post hoc comparisons of tetanus IgG geometric mean concentrations across age groups

| Age group | Age group | GMC mean difference in log-transformed IgG (95%CI) | Std. Error | *Adjusted P value* |
| --- | --- | --- | --- | --- |
| 1~5 month | 6~11 month | -0.756 (-0.944, -0.569) | 0.055 | <0.001 |
|  | 12~17 month | -0.693 (-0.878, -0.508) | 0.054 | <0.001 |
|  | 18~23 month | -0.724 (-0.909, -0.538) | 0.055 | <0.001 |
|  | 2 years | -0.741 (-0.923, -0.558) | 0.054 | <0.001 |
|  | 3 years | -0.660 (-0.853, -0.466) | 0.057 | <0.001 |
|  | 4 years | -0.545 (-0.745, -0.345) | 0.059 | <0.001 |
|  | 5 years | -0.539 (-0.733, -0.345) | 0.057 | <0.001 |
|  | 6 years | -0.642 (-0.837, -0.447) | 0.058 | <0.001 |
|  | 7~9 years | -0.488 (-0.676, -0.301) | 0.055 | <0.001 |
|  | 10~14 years | -0.260 (-0.451, -0.068) | 0.056 | 0.001 |
|  | 15~19 years | -0.135 (-0.331, 0.062) | 0.058 | 0.543 |
|  | 20~39 years | 0.218 (0.021, 0.415) | 0.058 | 0.015 |
|  | 40~59 years | 0.598 (0.410, 0.786) | 0.055 | <0.001 |
| 6~11 month | 1~5 month | 0.756 (0.569, 0.944) | 0.055 | <0.001 |
|  | 12~17 month | 0.063 (-0.013, 0.140) | 0.023 | 0.235 |
|  | 18~23 month | 0.032 (-0.047, 0.111) | 0.023 | 0.983 |
|  | 2 years | 0.015 (-0.055, 0.086) | 0.021 | 1 |
|  | 3 years | 0.096 (0.000, 0.193) | 0.029 | 0.052 |
|  | 4 years | 0.211 (0.101, 0.321) | 0.032 | <0.001 |
|  | 5 years | 0.217 (0.120, 0.315) | 0.029 | <0.001 |
|  | 6 years | 0.114 (0.014, 0.214) | 0.030 | 0.01 |
|  | 7~9 years | 0.268 (0.185, 0.350) | 0.025 | <0.001 |
|  | 10~14 years | 0.497 (0.405, 0.588) | 0.027 | <0.001 |
|  | 15~19 years | 0.621 (0.519, 0.724) | 0.031 | <0.001 |
|  | 20~39 years | 0.974 (0.871, 1.078) | 0.031 | <0.001 |
|  | 40~59 years | 1.354 (1.269, 1.439) | 0.025 | <0.001 |
| 12~17 month | 1~5 month | 0.693 (0.508, 0.878) | 0.054 | <0.001 |
|  | 6~11 month | -0.063 (-0.140, 0.013) | 0.023 | 0.235 |
|  | 18~23 month | -0.031 (-0.102, 0.041) | 0.021 | 0.976 |
|  | 2 years | -0.048 (-0.110, 0.015) | 0.018 | 0.356 |
|  | 3 years | 0.033 (-0.058, 0.124) | 0.027 | 0.994 |
|  | 4 years | 0.148 (0.043, 0.253) | 0.031 | <0.001 |
|  | 5 years | 0.154 (0.062, 0.246) | 0.027 | <0.001 |
|  | 6 years | 0.051 (-0.044, 0.145) | 0.028 | 0.866 |
|  | 7~9 years | 0.204 (0.129, 0.280) | 0.023 | <0.001 |
|  | 10~14 years | 0.433 (0.348, 0.519) | 0.025 | <0.001 |
|  | 15~19 years | 0.558 (0.461, 0.656) | 0.029 | <0.001 |
|  | 20~39 years | 0.911 (0.813, 1.009) | 0.029 | <0.001 |
|  | 40~59 years | 1.291 (1.213, 1.369) | 0.023 | <0.001 |
| 18~23 month | 1~5 month | 0.724 (0.538, 0.909) | 0.055 | <0.001 |
|  | 6~11 month | -0.032 (-0.111, 0.047) | 0.023 | 0.983 |
|  | 12~17 month | 0.031 (-0.041, 0.102) | 0.021 | 0.976 |
|  | 2 years | -0.017 (-0.082, 0.048) | 0.019 | 1 |
|  | 3 years | 0.064 (-0.029, 0.157) | 0.028 | 0.541 |
|  | 4 years | 0.179 (0.072, 0.285) | 0.032 | <0.001 |
|  | 5 years | 0.185 (0.091, 0.279) | 0.028 | <0.001 |
|  | 6 years | 0.082 (-0.015, 0.178) | 0.029 | 0.201 |
|  | 7~9 years | 0.235 (0.157, 0.314) | 0.023 | <0.001 |
|  | 10~14 years | 0.464 (0.377, 0.552) | 0.026 | <0.001 |
|  | 15~19 years | 0.589 (0.490, 0.688) | 0.029 | <0.001 |
|  | 20~39 years | 0.942 (0.842, 1.042) | 0.030 | <0.001 |
|  | 40~59 years | 1.322 (1.241, 1.403) | 0.024 | <0.001 |
| 2 years | 1~5 month | 0.741 (0.558, 0.923) | 0.054 | <0.001 |
|  | 6~11 month | -0.015 (-0.086, 0.055) | 0.021 | 1 |
|  | 12~17 month | 0.048 (-0.015, 0.110) | 0.018 | 0.356 |
|  | 18~23 month | 0.017 (-0.048, 0.082) | 0.019 | 1 |
|  | 3 years | 0.081 (-0.005, 0.167) | 0.026 | 0.091 |
|  | 4 years | 0.196 (0.095, 0.296) | 0.030 | <0.001 |
|  | 5 years | 0.202 (0.115, 0.289) | 0.026 | <0.001 |
|  | 6 years | 0.099 (0.009, 0.188) | 0.027 | 0.017 |
|  | 7~9 years | 0.252 (0.182, 0.322) | 0.021 | <0.001 |
|  | 10~14 years | 0.481 (0.401, 0.561) | 0.024 | <0.001 |
|  | 15~19 years | 0.606 (0.513, 0.699) | 0.028 | <0.001 |
|  | 20~39 years | 0.959 (0.866, 1.052) | 0.028 | <0.001 |
|  | 40~59 years | 1.339 (1.266, 1.411) | 0.022 | <0.001 |
| 3 years | 1~5 month | 0.660 (0.466, 0.853) | 0.057 | <0.001 |
|  | 6~11 month | -0.096 (-0.193, 0.000) | 0.029 | 0.052 |
|  | 12~17 month | -0.033 (-0.124, 0.058) | 0.027 | 0.994 |
|  | 18~23 month | -0.064 (-0.157, 0.029) | 0.028 | 0.541 |
|  | 2 years | -0.081 (-0.167, 0.005) | 0.026 | 0.091 |
|  | 4 years | 0.115 (-0.006, 0.235) | 0.036 | 0.08 |
|  | 5 years | 0.121 (0.011, 0.230) | 0.032 | 0.016 |
|  | 6 years | 0.018 (-0.094, 0.129) | 0.033 | 1 |
|  | 7~9 years | 0.171 (0.075, 0.268) | 0.029 | <0.001 |
|  | 10~14 years | 0.400 (0.296, 0.504) | 0.031 | <0.001 |
|  | 15~19 years | 0.525 (0.411, 0.639) | 0.034 | <0.001 |
|  | 20~39 years | 0.878 (0.764, 0.992) | 0.034 | <0.001 |
|  | 40~59 years | 1.258 (1.159, 1.356) | 0.029 | <0.001 |
| 4 years | 1~5 month | 0.545 (0.345, 0.745) | 0.059 | <0.001 |
|  | 6~11 month | -0.211 (-0.321, -0.101) | 0.032 | <0.001 |
|  | 12~17 month | -0.148 (-0.253, -0.043) | 0.031 | <0.001 |
|  | 18~23 month | -0.179 (-0.285, -0.072) | 0.032 | <0.001 |
|  | 2 years | -0.196 (-0.296, -0.095) | 0.030 | <0.001 |
|  | 3 years | -0.115 (-0.235, 0.006) | 0.036 | 0.08 |
|  | 5 years | 0.006 (-0.115, 0.127) | 0.036 | 1 |
|  | 6 years | -0.097 (-0.220, 0.026) | 0.036 | 0.301 |
|  | 7~9 years | 0.057 (-0.053, 0.166) | 0.032 | 0.898 |
|  | 10~14 years | 0.286 (0.170, 0.402) | 0.034 | <0.001 |
|  | 15~19 years | 0.410 (0.285, 0.536) | 0.037 | <0.001 |
|  | 20~39 years | 0.763 (0.638, 0.889) | 0.037 | <0.001 |
|  | 40~59 years | 1.143 (1.032, 1.254) | 0.033 | <0.001 |
| 5 years | 1~5 month | 0.539 (0.345, 0.733) | 0.057 | <0.001 |
|  | 6~11 month | -0.217 (-0.315, -0.120) | 0.029 | <0.001 |
|  | 12~17 month | -0.154 (-0.246, -0.062) | 0.027 | <0.001 |
|  | 18~23 month | -0.185 (-0.279, -0.091) | 0.028 | <0.001 |
|  | 2 years | -0.202 (-0.289, -0.115) | 0.026 | <0.001 |
|  | 3 years | -0.121 (-0.230, -0.011) | 0.032 | 0.016 |
|  | 4 years | -0.006 (-0.127, 0.115) | 0.036 | 1 |
|  | 6 years | -0.103 (-0.215, 0.009) | 0.033 | 0.108 |
|  | 7~9 years | 0.051 (-0.047, 0.148) | 0.029 | 0.894 |
|  | 10~14 years | 0.279 (0.175, 0.384) | 0.031 | <0.001 |
|  | 15~19 years | 0.404 (0.290, 0.519) | 0.034 | <0.001 |
|  | 20~39 years | 0.757 (0.642, 0.872) | 0.034 | <0.001 |
|  | 40~59 years | 1.137 (1.038, 1.236) | 0.029 | <0.001 |
| 6 years | 1~5 month | 0.642 (0.447, 0.837) | 0.058 | <0.001 |
|  | 6~11 month | -0.114 (-0.214, -0.014) | 0.030 | 0.01 |
|  | 12~17 month | -0.051 (-0.145, 0.044) | 0.028 | 0.866 |
|  | 18~23 month | -0.082 (-0.178, 0.015) | 0.029 | 0.201 |
|  | 2 years | -0.099 (-0.188, -0.009) | 0.027 | 0.017 |
|  | 3 years | -0.018 (-0.129, 0.094) | 0.033 | 1 |
|  | 4 years | 0.097 (-0.026, 0.220) | 0.036 | 0.301 |
|  | 5 years | 0.103 (-0.009, 0.215) | 0.033 | 0.108 |
|  | 7~9 years | 0.154 (0.054, 0.253) | 0.029 | <0.001 |
|  | 10~14 years | 0.383 (0.276, 0.489) | 0.032 | <0.001 |
|  | 15~19 years | 0.507 (0.391, 0.624) | 0.035 | <0.001 |
|  | 20~39 years | 0.860 (0.743, 0.977) | 0.035 | <0.001 |
|  | 40~59 years | 1.240 (1.139, 1.342) | 0.030 | <0.001 |
| 7~9 years | 1~5 month | 0.488 (0.301, 0.676) | 0.055 | <0.001 |
|  | 6~11 month | -0.268 (-0.350, -0.185) | 0.025 | <0.001 |
|  | 12~17 month | -0.204 (-0.280, -0.129) | 0.023 | <0.001 |
|  | 18~23 month | -0.235 (-0.314, -0.157) | 0.023 | <0.001 |
|  | 2 years | -0.252 (-0.322, -0.182) | 0.021 | <0.001 |
|  | 3 years | -0.171 (-0.268, -0.075) | 0.029 | <0.001 |
|  | 4 years | -0.057 (-0.166, 0.053) | 0.032 | 0.898 |
|  | 5 years | -0.051 (-0.148, 0.047) | 0.029 | 0.894 |
|  | 6 years | -0.154 (-0.253, -0.054) | 0.029 | <0.001 |
|  | 10~14 years | 0.229 (0.138, 0.320) | 0.027 | <0.001 |
|  | 15~19 years | 0.354 (0.251, 0.456) | 0.030 | <0.001 |
|  | 20~39 years | 0.707 (0.604, 0.809) | 0.031 | <0.001 |
|  | 40~59 years | 1.087 (1.002, 1.171) | 0.025 | <0.001 |
| 10~14 years | 1~5 month | 0.260 (0.068, 0.451) | 0.056 | 0.001 |
|  | 6~11 month | -0.497 (-0.588, -0.405) | 0.027 | <0.001 |
|  | 12~17 month | -0.433 (-0.519, -0.348) | 0.025 | <0.001 |
|  | 18~23 month | -0.464 (-0.552, -0.377) | 0.026 | <0.001 |
|  | 2 years | -0.481 (-0.561, -0.401) | 0.024 | <0.001 |
|  | 3 years | -0.400 (-0.504, -0.296) | 0.031 | <0.001 |
|  | 4 years | -0.286 (-0.402, -0.170) | 0.034 | <0.001 |
|  | 5 years | -0.279 (-0.384, -0.175) | 0.031 | <0.001 |
|  | 6 years | -0.383 (-0.489, -0.276) | 0.032 | <0.001 |
|  | 7~9 years | -0.229 (-0.320, -0.138) | 0.027 | <0.001 |
|  | 15~19 years | 0.125 (0.015, 0.234) | 0.033 | 0.01 |
|  | 20~39 years | 0.478 (0.368, 0.588) | 0.033 | <0.001 |
|  | 40~59 years | 0.858 (0.764, 0.951) | 0.028 | <0.001 |
| 15~19 years | 1~5 month | 0.135 (-0.062, 0.331) | 0.058 | 0.543 |
|  | 6~11 month | -0.621 (-0.724, -0.519) | 0.031 | <0.001 |
|  | 12~17 month | -0.558 (-0.656, -0.461) | 0.029 | <0.001 |
|  | 18~23 month | -0.589 (-0.688, -0.490) | 0.029 | <0.001 |
|  | 2 years | -0.606 (-0.699, -0.513) | 0.028 | <0.001 |
|  | 3 years | -0.525 (-0.639, -0.411) | 0.034 | <0.001 |
|  | 4 years | -0.410 (-0.536, -0.285) | 0.037 | <0.001 |
|  | 5 years | -0.404 (-0.519, -0.290) | 0.034 | <0.001 |
|  | 6 years | -0.507 (-0.624, -0.391) | 0.035 | <0.001 |
|  | 7~9 years | -0.354 (-0.456, -0.251) | 0.030 | <0.001 |
|  | 10~14 years | -0.125 (-0.234, -0.015) | 0.033 | 0.01 |
|  | 20~39 years | 0.353 (0.234, 0.472) | 0.035 | <0.001 |
|  | 40~59 years | 0.733 (0.628, 0.837) | 0.031 | <0.001 |
| 20~39 years | 1~5 month | -0.218 (-0.415, -0.021) | 0.058 | 0.015 |
|  | 6~11 month | -0.974 (-1.078, -0.871) | 0.031 | <0.001 |
|  | 12~17 month | -0.911 (-1.009, -0.813) | 0.029 | <0.001 |
|  | 18~23 month | -0.942 (-1.042, -0.842) | 0.030 | <0.001 |
|  | 2 years | -0.959 (-1.052, -0.866) | 0.028 | <0.001 |
|  | 3 years | -0.878 (-0.992, -0.764) | 0.034 | <0.001 |
|  | 4 years | -0.763 (-0.889, -0.638) | 0.037 | <0.001 |
|  | 5 years | -0.757 (-0.872, -0.642) | 0.034 | <0.001 |
|  | 6 years | -0.860 (-0.977, -0.743) | 0.035 | <0.001 |
|  | 7~9 years | -0.707 (-0.809, -0.604) | 0.031 | <0.001 |
|  | 10~14 years | -0.478 (-0.588, -0.368) | 0.033 | <0.001 |
|  | 15~19 years | -0.353 (-0.472, -0.234) | 0.035 | <0.001 |
|  | 40~59 years | 0.380 (0.275, 0.485) | 0.031 | <0.001 |
| 40~59 years | 1~5 month | -0.598 (-0.786, -0.410) | 0.055 | <0.001 |
|  | 6~11 month | -1.354 (-1.439, -1.269) | 0.025 | <0.001 |
|  | 12~17 month | -1.291 (-1.369, -1.213) | 0.023 | <0.001 |
|  | 18~23 month | -1.322 (-1.403, -1.241) | 0.024 | <0.001 |
|  | 2 years | -1.339 (-1.411, -1.266) | 0.022 | <0.001 |
|  | 3 years | -1.258 (-1.356, -1.159) | 0.029 | <0.001 |
|  | 4 years | -1.143 (-1.254, -1.032) | 0.033 | <0.001 |
|  | 5 years | -1.137 (-1.236, -1.038) | 0.029 | <0.001 |
|  | 6 years | -1.240 (-1.342, -1.139) | 0.030 | <0.001 |
|  | 7~9 years | -1.087 (-1.171, -1.002) | 0.025 | <0.001 |
|  | 10~14 years | -0.858 (-0.951, -0.764) | 0.028 | <0.001 |
|  | 15~19 years | -0.733 (-0.837, -0.628) | 0.031 | <0.001 |
|  | 20~39 years | -0.380 (-0.485, -0.275) | 0.031 | <0.001 |

Note: GMC, geometric mean concentration. Pairwise comparisons were performed using log-transformed tetanus IgG antibody concentrations. Games-Howell post hoc tests were used because of unequal variances across groups.

Supplementary Table S2. Pairwise post hoc comparisons of tetanus IgG geometric mean concentrations across documented vaccine dose groups

| dose | dose | GMC mean difference in log-transformed IgG (95%CI) | Std. Error | *Adjusted P value* |
| --- | --- | --- | --- | --- |
| 0 | 1 | -0.638 (-0.926, -0.349) | 0.097 | <0.001 |
|  | 2 | -1.073 (-1.192, -0.954) | 0.041 | <0.001 |
|  | 3 | -1.080 (-1.144, -1.016) | 0.023 | <0.001 |
|  | 4 | -0.979 (-1.044, -0.914) | 0.023 | <0.001 |
|  | 5 | -0.675 (-0.745, -0.604) | 0.025 | <0.001 |
| 1 | 0 | 0.638 (0.349, 0.926) | 0.097 | <0.001 |
|  | 2 | -0.435 (-0.735, -0.136) | 0.101 | 0.001 |
|  | 3 | -0.442 (-0.727, -0.158) | 0.095 | 0.001 |
|  | 4 | -0.341 (-0.626, -0.057) | 0.095 | 0.011 |
|  | 5 | -0.037 (-0.323, 0.249) | 0.096 | 0.999 |
| 2 | 0 | 1.073 (0.954, 1.192) | 0.041 | <0.001 |
|  | 1 | 0.435 (0.136, 0.735) | 0.101 | 0.001 |
|  | 3 | -0.007 (-0.115, 0.101) | 0.037 | 1 |
|  | 4 | 0.094 (-0.015, 0.203) | 0.037 | 0.133 |
|  | 5 | 0.398 (0.286, 0.510) | 0.039 | <0.001 |
| 3 | 0 | 1.080 (1.016, 1.144) | 0.023 | <0.001 |
|  | 1 | 0.442 (0.158, 0.727) | 0.095 | 0.001 |
|  | 2 | 0.007 (-0.101, 0.115) | 0.037 | 1 |
|  | 4 | 0.101 (0.059, 0.142) | 0.015 | <0.001 |
|  | 5 | 0.405 (0.356, 0.455) | 0.017 | <0.001 |
| 4 | 0 | 0.979 (0.914, 1.044) | 0.023 | <0.001 |
|  | 1 | 0.341 (0.057, 0.626) | 0.095 | 0.011 |
|  | 2 | -0.094 (-0.203, 0.015) | 0.037 | 0.133 |
|  | 3 | -0.101 (-0.142, -0.059) | 0.015 | <0.001 |
|  | 5 | 0.304 (0.254, 0.355) | 0.018 | <0.001 |
| 5 | 0 | 0.675 (0.604, 0.745) | 0.025 | <0.001 |
|  | 1 | 0.037 (-0.249, 0.323) | 0.096 | 0.999 |
|  | 2 | -0.398 (-0.510, -0.286) | 0.039 | <0.001 |
|  | 3 | -0.405 (-0.455, -0.356) | 0.017 | <0.001 |
|  | 4 | -0.304 (-0.355, -0.254) | 0.018 | <0.001 |

Note: GMC, geometric mean concentration. Pairwise comparisons were performed using log-transformed tetanus IgG antibody concentrations. Games-Howell post hoc tests were used because of unequal variances across groups.

Supplementary Table S3. Age distribution, GMC, sex, residence, time since the last dose, seropositivity, and seroprotection by dose group based on system verification vaccination.

| Variable | Dose | | |
| --- | --- | --- | --- |
|  | 3 | 4 | 5 |
| Number | 462 | 1146 | 947 |
| GMC (95%CI) | 0.170 (0.165, 0.175) | 0.156 (0.152, 0.161) | 0.108 (0.103, 0.114) |
| ≥0.01IU/ml,n | 460 | 1121 | 914 |
| ≥0.1IU/ml,n | 416 | 879 | 440 |
| Age, mean ± SD | 1.42±1.57 | 4.56±3.34 | 10.73±3.78 |
| Time since the last dose, mean ± SD | 0.89±1.50 | 2.89±3.25 | 4.56±3.74 |
| Sex |  |  |  |
| Male | 272 | 626 | 488 |
| Female | 190 | 520 | 459 |
| Residence |  |  |  |
| Urban | 204 | 604 | 533 |
| Rural | 258 | 542 | 414 |

Note: GMC, geometric mean concentration. Pairwise comparisons were performed using log-transformed tetanus IgG antibody concentrations. Games-Howell post hoc tests were used because of unequal variances across groups. There was a statistically significant difference in GMC between dose 4 and dose 5, GMC mean difference in log-transformed IgG (95%CI):0.226 (0.179,0.272), *P*<0.001.

Supplementary Table S4. Concurvity diagnostics for the overall adjusted GAM.

| Model | Model component | Variables / terms included | Worst | Observed | Estimate | EDF | F | *P* |
| --- | --- | --- | --- | --- | --- | --- | --- | --- |
| Overall adjusted GAM | Smooth term | s(time since the last dose) | 0.266 | 0.224 | 0.062 | 4.384 | 58.31 | <0.001 |

Abbreviations: GAM, generalized additive model.

Model specification: Gamma family with log link; outcome variable was tetanus IgG antibody concentration. The overall adjusted model included a smooth term for time since vaccination and adjusted for sex and vaccine dose group.

Note: Concurvity values range from 0 to 1, with values closer to 1 indicating stronger nonlinear dependence between model terms. “Worst” represents the maximum possible concurvity, “Observed” represents concurvity for the fitted functions, and “Estimate” is a basis-function-based estimate. The parametric component was reported as a combined block by the model output.
