# Supplementary figures and images for: Population waning trajectories of vaccine-induced tetanus immunity in Zhejiang, China
Source: Front Immunol. 2026 May 25;17:1824381. doi: 10.3389/fimmu.2026.1824381 (PMC13243430; doi:10.3389/fimmu.2026.1824381)

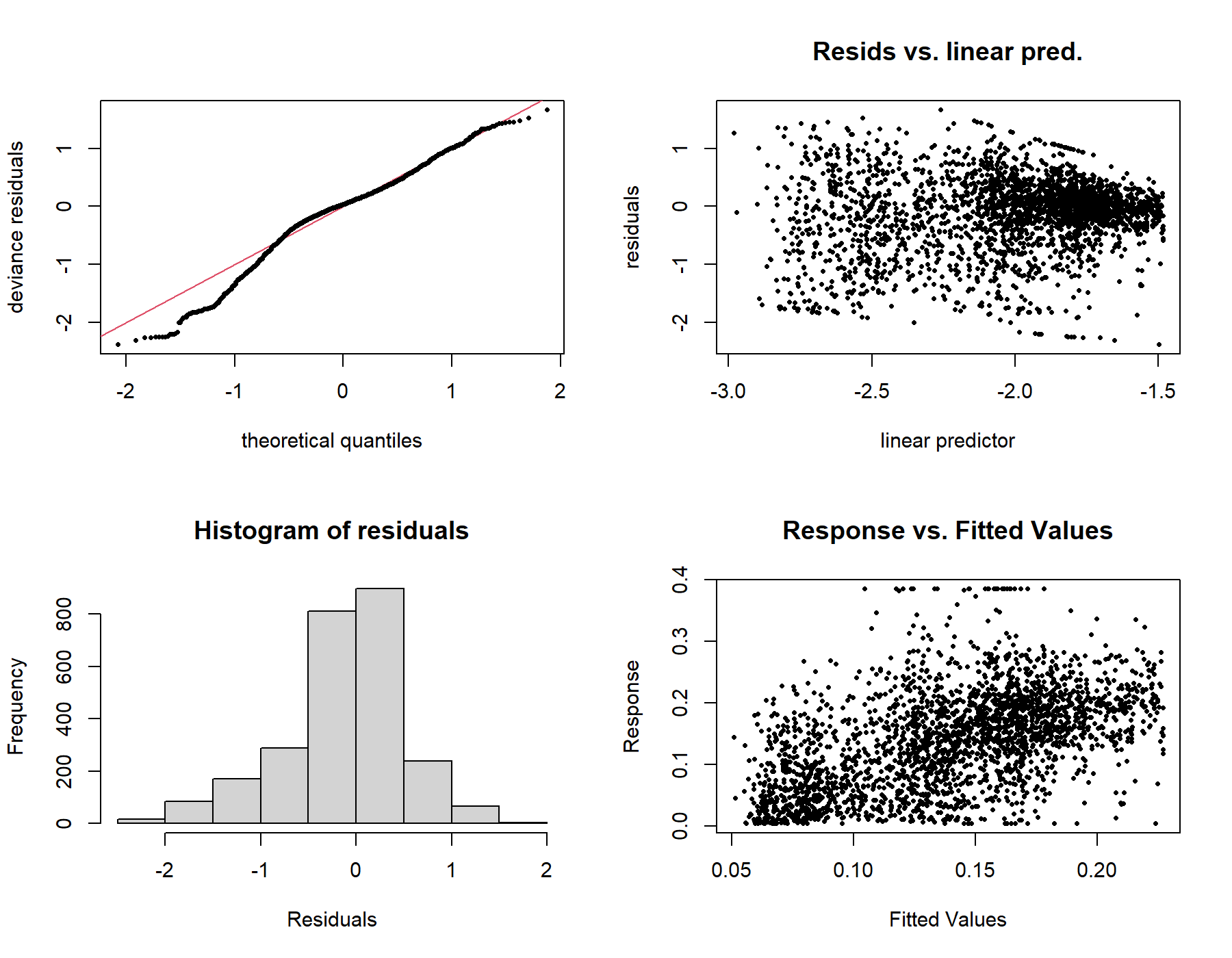

Supplement: Supplementary Figure 1 — Diagnostic plots for the overall adjusted generalized additive model (GAM). The plots show the quantile-quantile plot of deviance residuals, residuals versus the linear predictor, the histogram of residuals, and observed versus fitted values. Red reference lines indicate the expected pattern under adequate model fit. [file Image1.png]

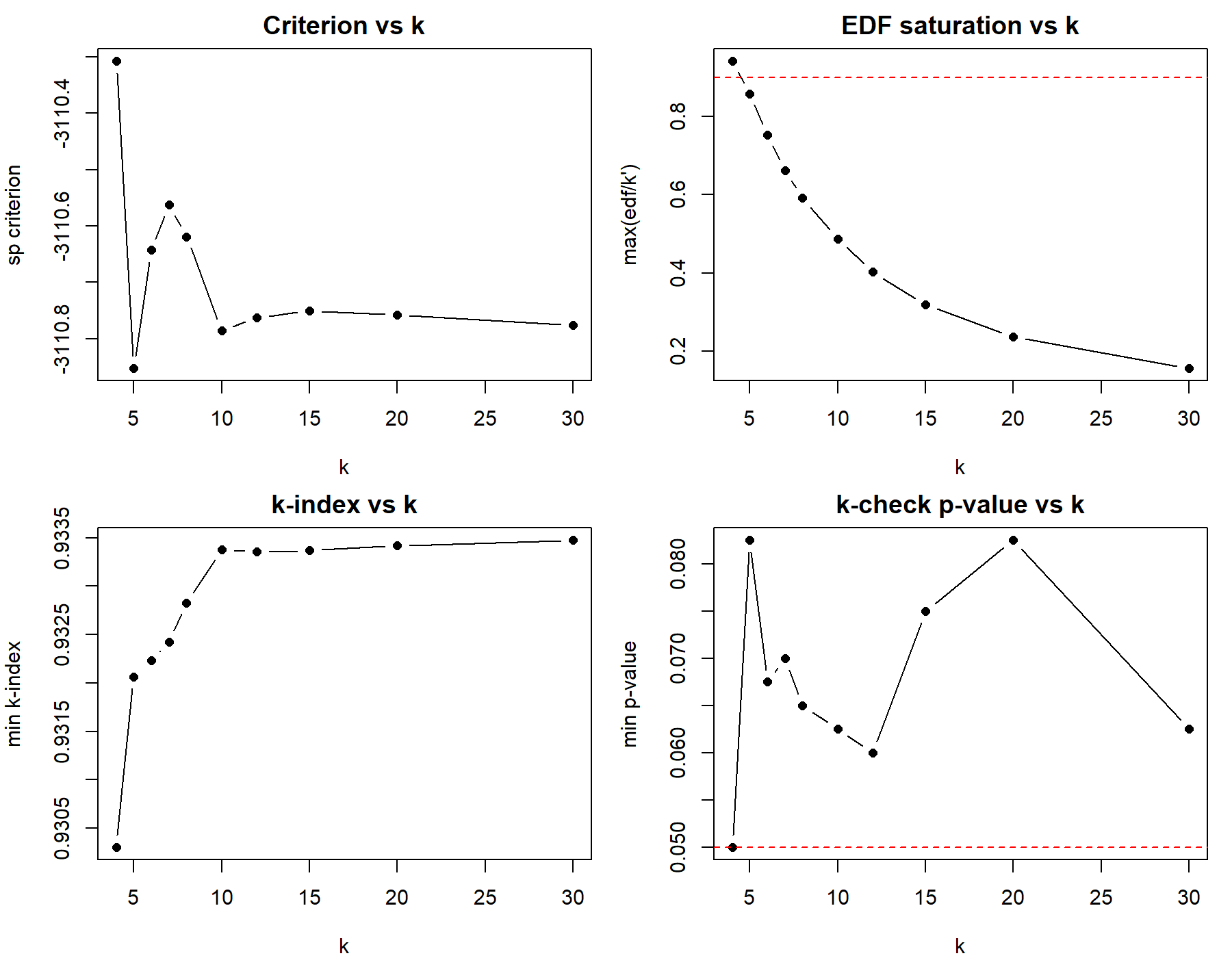

Supplement: Supplementary Figure 2 — Basis-dimension diagnostics for the overall adjusted generalized additive model (GAM). The plots show the smoothing criterion, EDF saturation, minimum k-index, and minimum k-check P value across candidate basis dimensions (k). Red dashed lines indicate reference thresholds. [file Image2.png]

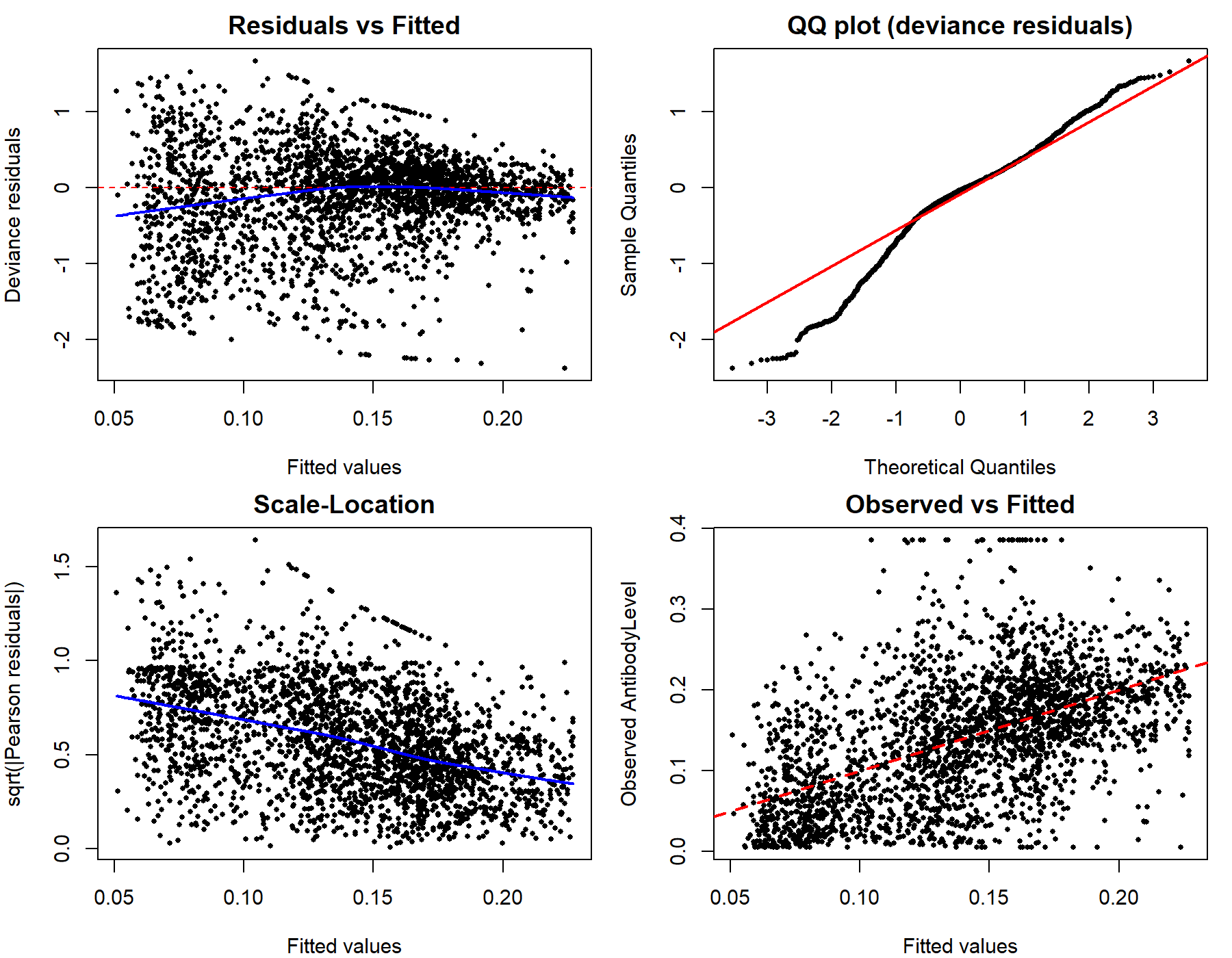

Supplement: Supplementary Figure 3 — Residual diagnostics for the overall adjusted generalized additive model (GAM). The plots show residuals versus fitted values, the QQ plot of deviance residuals, the scale–location plot, and observed versus fitted values. Blue smooth curves indicate local trends, and red dashed or solid lines indicate reference lines. [file Image3.png]
